# Supplementary material for: Secondary Malignancies after Ewing Sarcoma—Epidemiological and Clinical Analysis of an International Trial Registry
Source: Cancers (Basel). 2022 Nov 30;14(23):5920. doi: 10.3390/cancers14235920 (PMC9740851; doi:10.3390/cancers14235920)
Supplement: Supplementary file 1 [file cancers-14-05920-s001.zip › cancers-1968438-supplementary.pdf]

Supplementary Materials:

Secondary Malignancies after Ewing Sarcoma—Epidemiological and Clinical Analysis of an International Trial Registry

Isabelle Kaiser, Katja Kauertz, Stefan K. Zöllner, Wolfgang Hartmann, Thorsten Langer, Heribert Jürgens, Andreas Ranft and Uta Dirksen

Table S1. Patient characteristics and clinical features of 96 primary Ewing sarcoma (EwS) patients with > 1 subsequent malignant neoplasms (SMNs) in the CESS 81, CESS 86, EICESS 92, Euro-E.W.I.N.G. 99, and Ewing 2008 trials.

EwS trial (n = 5)	Number of Patients with SMNs (n, %)
CESS 81	0 (of 5), 0%
CESS 86	1 (of 5), 20%
EICESS 92	1 (of 5), 20%
EURO E.W.I.N.G. 99	0 (of 5), 0%
Ewing 2008	3 (of 5), 60%
Sex (%) (n = 5)	
Male	2 (40%)
Female	3 (60%)
Metastases (n = 5)	
Yes	2 (40%)
No	3 (60%)
Latency time (median (range))	
EwS to first SMNs	8.1 (0.4– 26.8) years
EwS to second SMNs	9.36 (0.7- 27) years
Localization (n = 96)	
Cranium	0 (0%)
Hand/foot	0 (0%)
Upper limb	1 (20%)
Lower limb	0 (0%)
Axial skeleton	2 (40%)
Pelvis	2 (40%)
